# Recent advances in Japanese encephalitis

**DOI:** 10.12688/f1000research.9561.1

**Published:** 2017-03-13

**Authors:** Anirban Basu, Kallol Dutta

**Affiliations:** 1 National Brain Research Centre, Manesar, Haryana, India; 2 Le Centre de recherche de l'Institut universitaire en santé mentale de Québec, Québec City, Canada

**Keywords:** Japanese encephalitis, flavivirus, microRNA, Minocycline, vaccine

## Abstract

Japanese encephalitis is a flaviviral disease that is endemic to the South, Southeast Asia, and Asia Oceania regions. Given that about 60% of the world’s population (about 7.4 billion) resides in this region (about 4.4 billion), this disease poses a significant threat to global health. Active vaccination campaigns conducted in endemic countries have led to a decrease in the number of reported cases over the years. In this article, we strive to briefly highlight recent advances in understanding the role of microRNAs in disease pathology, focus on providing brief summaries of recent clinical trials in the field of Japanese encephalitis therapeutics, and review the current prophylactic strategies.

## Ecology and epidemiology of Japanese encephalitis

Japanese encephalitis (JE) is a mosquito-borne flaviviral disease, primarily affecting children between the ages of 0 to 15 years and occasionally adults. The enzootic life cycle of the virus results in its transmission to vertebrate hosts by mosquitoes, mainly belonging to the *Culex* sp. Ardeid birds and bats serve as virus reservoirs. Humans are “dead end hosts” as the virus cannot be transmitted from one infected person to another ^1^. Pigs serve as amplification hosts as the virus aggressively replicates, resulting in viremia with a very high viral titer. Usually, infected pig–to–non-infected pig transmission was thought to be carried out by the mosquitoes. However, a recent investigation reports that viral transfer between pigs could be independent of the vector. It was reported that oronasal secretion of pigs contains the JE virus (JEV) at sufficiently high titers so as to infect another naïve animal ^2^. This could explain the viral persistence in pigs during winter, when there is a decrease in the vector population. This also means that vector control would not be a sufficient measure to arrest the spread of the disease in cases of outbreak. According to the World Health Organization (WHO), annually there are about 67,900 global cases of JE, of which 20–30% are fatal, and 30–50% of survivors have significant neurological sequelae ^3^. The current endemic region of JE encompasses the entire South, Southeast Asia, eastern parts of the Russian Federation, parts of Australia, and a few Western Pacific islands such as Saipan and Papua New Guinea. In cases of human infection, the virus rapidly infects the central nervous system (CNS), resulting in severe neuroinflammation and ultimately neuronal death. Concomitantly, JEV disrupt the neural stem/progenitor cell pool in the germinal niches of the CNS and their efficacy at generating functional neurons, thereby stalling the neuronal repair ^4^.

## MicroRNAs: the new players in the field

MicroRNAs (miRNAs) are small (about 22-nucleotide) non-coding RNAs that are involved in RNA-directed gene expression regulation in a wide spectrum of biological systems. Viruses are known to manipulate host miRNA expression for their replication, propagation, or immune evasion ^5^. Flaviviruses are known to generate viral sub-genomic RNAs in infected cells and to modulate cellular miRNAs ^6, 7^. The earliest studies relating miRNAs and flaviviruses primarily focused on attenuation of neuro-virulence of the viruses by insertion of miRNA targets within the viral genome, thereby making them ideal candidates for vaccine development ^8, 9^. A couple of years ago, we first reported a causal role of miRNAs in JEV-induced neuroinflammation. We observed that miRNA-29b was significantly increased in mouse microglial cells following infection. MiRNA-29b was predicted to target tumor necrosis factor alpha–induced protein 3, a negative regulator of the nuclear factor kappa B (NFκB) signaling pathway. As microglia is involved in modulating CNS inflammation, our study suggested a role of miRNA-29b in such processes ^10^. Later, miRNA-155 was also identified to stimulate the NFκB pathway through activation of TANK-binding kinase 1 (TBK-1) via inhibition of SHIP1 protein in cultured mouse and human microglial cells as well as in brain samples ^11^. However, somewhat contradictory to this finding, another study reported that in the human microglial cell line, induction of miRNA-155 reduced JEV-induced innate immune gene expression and probably limited viral replication ^12^. MiRNA-146a was found to be overexpressed in microglia during JEV infection, leading to suppression of NFκB activity and inhibition of the anti-viral JAK-STAT pathway, thus helping the virus to evade the host’s innate immune mechanism. Interestingly, this mechanism seemed to be virus strain–specific; whereas the JaOArS982 strain of the virus led to induction of miRNA-146a, the P20778 strain had the exact contradictory effect in the same model ^13^. Another study reported that miRNA-15b is overexpressed in microglial cells and, in mouse brain as a whole during JEV infection, inhibited ring finger protein 125 (a negative regulator of RIG-I signaling), thereby leading to a higher production of pro-inflammatory cytokines and type I interferons. Knockdown of virus-induced miRNA-15b attenuated the pro-inflammatory response and had multiple pro-survival effects in the JEV-infected mouse model ^14^. Mechanistically, it was shown that the virus-induced expression of miRNA-15b is modulated by NFκB subunit c-Rel and cAMP-response element-binding protein (CREB) in response to JEV infection ^15^. Similarly, miRNA-19b-3p has been reported to positively regulate the JEV-induced inflammatory response in mouse brain and specifically glial cells by targeting ring finger protein 11, a negative regulator of NFκB signaling. Inhibition of ring finger protein 11 by miRNA-19b-3p activated NFκB pathway, which in turn led to higher production of inflammatory cyto/chemokines during infection ^16^. MiRNA-432 and miRNA-370 are examples of miRNAs that are downregulated in mouse brain and microglial cell lines following infection with JEV ^17, 18^. The *SOCS5* gene is negatively regulated by miRNA-432, and during JEV infection *SOCS5* is upregulated, leading to negative regulation of the anti-viral JAK-STAT pathway which helps in viral immune evasion. MiRNA-370 has been suggested to negatively regulate NFκB-mediated inflammatory pathways and interferon production. Thus, JEV-induced downregulation of this miRNA would facilitate inflammatory and anti-viral pathways in microglial cells. However, in *in vitro* conditions, a miR-370 mimic was found to inhibit replication of JEV, which is contrary to its proposed mechanism of action ^18^. JEV infection of HEK293 (human embryonic kidney cell line) resulted in downregulation of miRNA-33a-5p, which acts as a regulator of eukaryotic translation elongation factor 1A1 (EEF1A1). EEF1A1 binds to and stabilizes the viral replicase complex consisting of the NS3 and NS5 proteins, thereby facilitating JEV replication ^19^. Another recent study reported the role of miRNA-124 in JEV infection ^20^. MiRNA-124 is highly expressed in neurons and has recently been reported to be one of many (113) miRNAs to be differentially expressed in porcine cells infected with JEV ^21^. Its expression is upregulated in JEV-infected swine testis cells. Overexpression of miRNA-124 in PK15 (porcine kidney 15) cells resulted in inhibition of replication of the infecting JEV. MiRNA-124 was found to target the dynamin 2 ( *DNM2*) gene of the host cell which produces a GTPase responsible for vesicle scission. MiRNA-124 possibly negatively regulates DNM2 production, thereby depriving access to the cellular membrane vesicular system which is required for viral replication and propagation ^22, 23^ and thus is part of the host response aimed at limiting JEV infection. Another miRNA that is likely to be involved in the regulation of neuroinflammation following JEV infection is miRNA-22. MiRNA-22 has recently been reported to be induced in glial cells after treatment with polyinosinic:polycytidylic acid (poly I:C) (double-stranded RNA mimetic) which potentially results in inhibition of pro-inflammatory cyto/chemokines and suppresses anti-viral interferon responses by acting upon mitochondrial anti-viral signaling protein (MAVS) ^24^. Interestingly, MAVS lies downstream of the RIG-I-STING pathway of JEV sensing which leads to activation of interferon responses ^25, 26^. Thus, any JEV-induced upregulation of miRNA-22 would facilitate survival of the virus in host cells. In this context, it should be noted that type 1 interferon response has been shown to impart protection against flavivirus (including JEV)-mediated cytotoxicity in glial cells ^27^.

Subsequent to the identification of roles that these miRNAs play in regulating inflammation or viral replication, investigations have been carried out to identify miRNAs that are differentially expressed in a particular cell type or organ following JEV infection and the pathways affected by them ^18, 21, 28– 30^. These investigations have led to detection of more than a thousand different miRNAs (known or novel) to be differentially expressed in JE. The genes or pathways affected by these are being worked out; some of them are pathways in cancer, the neurotrophin signaling pathway, the Toll-like receptor signaling pathway, the Notch signaling pathways, and the JAK-STAT signaling pathway. In exosomes isolated from cerebrospinal fluid of JEV-infected human subjects, miR-21-5p, miR-150-5p, and miR-342-3p levels were found to be elevated, a trend that is also observed in infected mice brain ^31^. Bioinformatic analysis for putative target genes of these three miRNAs indicated the involvement of transforming growth factor-beta (TGF-β), nerve growth factor (NGF), axon guidance, and mitogen-activated protein kinase (MAPK) signaling pathways ^31^. Thus, a vista of novel information has begun to be available that would help in better understanding the molecular pathology of JE and hopefully also in developing newer therapeutic interventions.

## Therapeutic approach to Japanese encephalitis: dark clouds with silver linings

More than a century of research has not been able to develop an effective therapeutic countermeasure to tackle JE (or any other flavivirus for that matter). In many cases, promising drugs/molecules that have been found to be efficacious in *in vitro* or *in vivo* animal models either were not deemed suitable for or failed to replicate their success in actual human trials. Supplementary Table 1 (an updated version of a similar table in a book chapter by the same authors ^32^) gives a detailed list of all those drugs/molecules that have been reported to date to counter JE in various models of the disease. Clinical trials involving administration of dexamethasone ^33^, interferon alpha 2a ^34^, and ribavirin ^35^ were not successful. However, three recent trials with two different drugs or molecules hold promise for further investigations. The first of them is minocycline, a second-generation tetracycline antibiotic whose protective role in JE has been investigated and reported by our group during the last 8 years ^36– 41^. A concise description of the probable mechanisms of action of minocycline can be found in a review we authored a few years ago ^42^. A culmination of those efforts was the first randomized placebo-controlled clinical trial of minocycline administration to patients with JE conducted in the pediatrics department of King George’s Medical University, in Lucknow, in the Indian state of Uttar Pradesh. Owing to logistic constraints, the subject recruitment criteria encompassed all cases falling under acute encephalitis syndrome (AES) ^43^. AES is characterized by abrupt onset of fever and often is accompanied with headache and vomiting followed by convulsions. Thus, in many cases when the patients were hospitalized or enrolled for the trial, they were in a coma and were hemodynamically unstable. Minocycline (or placebo) was administered as suspension through a nasogastric tube for 7 days at a loading dose of 5 mg/kg per day followed by 2.5 mg/kg every 12 hours in children up to 12 years old and a 200 mg loading dose followed by 100 mg every 12 hours in older patients. A total of 140 patients received minocycline and 141 received placebo. Even though there was not a statistically significant difference in survival between the drug and placebo groups, when the Glasgow Outcome Score at 3 months after discharge was compared between survivors of the two groups, there was a clear significant improvement with minocycline. Moreover, if the data from patients who succumbed within 24 hours of hospitalization/enrollment were excluded, then significantly better overall outcome was observed at 3 months in those receiving minocycline along with a trend toward lower cumulative mortality.

The second trial with minocycline was conducted in the Baba Raghav Das Medical College, in Gorakhpur, also in the Indian state of Uttar Pradesh, on a much smaller population; only 44 patients were enrolled. However, this study included only confirmed cases of JE. The dosage of minocycline used for this study was 5 to 6 mg/kg in two divided doses administered for 10 days through a feeding tube, which was started from the day of hospitalization/enrollment. At the conclusion of the trial, it was observed that minocycline was effective in reducing the duration of symptoms like fever and unconsciousness and the mean duration of hospitalization. However, owing to the small sample size and the availability of advanced life support and the early referral facility of patients from remote areas, decreased mortality and increased full clinical recovery observed in the drug-treated group could not be statistically correlated with treatment alone ^44^. The other significant difference of this study from the previous one is in the methodology used to evaluate neurological deficits and behavioral outcomes. Whereas the earlier study used the Glasgow outcome scoring ^45^, this study used the Liverpool outcome scoring, devised specifically to assess neurological disability in patients with JE ^46^.

The third report was from a feasibility study conducted in Nepal, involving administration of intravenous immunoglobulin (IVIG) to patients with JE ^47^. Theoretically, IVIG purified from pooled plasma of healthy donors from JE-endemic zones would have high titers of specific neutralizing antibody, as a large part of the population is postulated to have been exposed to the virus, and thus have antibodies ^48, 49^. In fact, IVIG that is not hyper-immune to JEV has already been reported to impart therapeutic benefits in the recovery from JE ^50^. This concept has also been adopted for West Nile virus infection with some success ^51, 52^. A comparison was done between IVIG collected from four different commercial sources (two from India and two from China), and the one that showed maximum 50% plaque reduction neutralization titers against wild-type JEV infecting a standard culture of Vero cells was chosen for the study. A small group of children (11 per group; ages between 1 and 14 years) manifesting symptoms of AES was recruited for the study and randomly divided into IVIG and placebo groups. IVIG administration is an onerous process requiring trained personnel and constant monitoring. It is delivered using a syringe driver, initially at a low infusion rate, which can be increased over time if the treatment is well tolerated. In this study, the AES-affected patients received either IVIG at a dose of 400 mg/kg per day for 5 days or an equivalent volume of 0.9% normal saline. The initial infusion rate was kept at 0.01 to 0.02 mL/kg body weight/minute and, if well tolerated, was gradually increased over 30 to 60 minutes to a maximum rate of 0.08 mL/kg body weight/minute. At hospital discharge, most of the patients belonging to either group demonstrated major sequelae; at 3- to 6-month follow-up, 45% in the IVIG group and 18% in the placebo group exhibited complete recovery (no neurological sequelae). However, no significant difference was observed between the two groups when analyzed by intention-to-treat to determine the proportion of patients exhibiting complete recovery either at hospital discharge or at follow-up. JEV neutralizing antibody titers were expectedly higher in patients who received IVIG compared with placebo. The other interesting aspect of IVIG is its ability to induce anti-inflammatory responses in the subjects non-specifically, usually by suppression of various pro-inflammatory mediators, including cyto/chemokines, and metalloproteinases ^53^. In this trial, the investigators report that the level of interleukin-4 (IL-4) was found to be significantly elevated in IVIG-treated patients. IL-4 is a complex cytokine that affects various regulatory pathways ^54^ and its higher levels have been detected in survivors of JE as compared with non-survivors ^55^. Thus, in summary, it can be said that all three trials, despite their limitations, do provide hope for the future when we might be able to counter JE therapeutically.

Apart from drugs that target the virus or disease pathologies, those that could possibly boost the host’s endogenous anti-viral mechanisms could also provide a novel approach to anti-viral therapy. As indicated earlier, type I interferons (IFN-α/β) are potent anti-viral proteins synthesized as a response to viral infection and lead to the production of a broad range of anti-viral proteins and immunoactive cytokines ^56^. The 2′,5′-oligoadenylate synthetase is one such protein that has been reported to be effective in inhibiting flaviviral replication ^57^. An important class of interferon-induced proteins are the members of the tripartite motif-containing (TRIM) protein superfamily that are now known to be involved in a broad range of biological processes associated with innate immunity. Recently, TRIM52, a unique member of the C-V sub-family of TRIM proteins, was reported to impart anti-JEV activity by targeting and degrading JEV non-structural protein 2A, a part of the viral replication complex ^58^. Another novel therapeutic approach could involve the CRISPR/Cas9 system to target a specific nucleotide sequence of the viral genome. This technique has already been successfully tried in experimental conditions on some human viruses and soon may be part of a comprehensive therapy for viral infections ^59^.

## Prevention is better than cure

Immunization policy against JEV has been implemented in most of the countries in the endemic zone, and as a result, there has been a decline in the number of cases and the fatality ratio due to the disease. The first-generation vaccines that were used were mouse brain-derived and were made from either Nakayama or Beijing-1 virus strains. These were highly efficacious and extensively employed in multiple countries for mass immunization. However, uncertainty over duration of protection, requirement of multiple booster doses, and rare reports of acute disseminated encephalomyelitis temporally associated with this type of vaccine resulted in the search for a newer generation of vaccines with a better safety profile. The current crop of JE vaccines are either inactivated Vero (African green monkey kidney epithelial) cell-based (JEBIK V, ENCEVAC, JEVAC, IXIARO/JESPECT, JEEV, and JENVAC) or PHK (primary hamster kidney) cell-based live attenuated (SA-14-14-2 CD.JEVAX) vaccines. The fourth type that is currently available is a recombinant chimeric virus vaccine. It was developed using the YFV17D vaccine vector of Yellow fever virus by replacing the cDNA encoding the envelope proteins of YFV with that of an attenuated SA14-14-2 strain of JEV (IMOJEV). This vaccine has also been found to be safe for use as a booster dose in children who have been previously immunized with the live attenuated vaccine ^60^. A detailed description of the characteristics, properties, and required dosages of these vaccines is available from the background paper on JE vaccines published by the Strategic Advisory Group of Experts of the WHO in 2014 ^61^. A concise idea can be generated from Appendix 4 of the same report. Based on the model of recombinant chimeric virus vaccine, a new recombinant modified vaccinia virus (Ankara) vaccine expressing JEV prM/E has been recently reported to be efficacious in mice models. Interestingly, when this vaccine was administered via a sub-lingual route, it elicited a protective immune response comparable to the one administered by the usual intramuscular route ^62^. A similar approach involving baculovirus vectors and subsequent immunization of mice also imparted protection against infection ^63^.

Among the various vaccines currently available, IMOJEV and SA-14-14-2 CD.JEVAX in single doses have been shown to elicit identical seroconversion and seroprotection rates ^64^; a single dose of IMOJEV also induced protective immunity similar to that induced in adults by three doses of a mouse brain-derived inactivated JE vaccine ^65^. Another study comparing usage of JENVAC (two doses, 28 days apart) and SA-14-14-2 CD.JEVAX (one dose) showed greater seroconversion and seroprotection in case of immunization with the former ^66^. However, this kind of comparison between an inactivated vaccine and a live attenuated vaccine is necessarily skewed and thus cannot be considered to be the basis for efficacy.

In spite of effective vaccine availability in almost all of the countries coming under the endemic region, there are reported cases of JE every year from many of them. Lack of awareness about the disease and lax immunization schedules could be blamed to a certain extent ^67^. Moreover, there is a possibility of JE affecting adults, especially in virgin areas, where there is low natural immunity against the virus ^68, 69^. Another cause of concern is the emergence of other genotypes of JEV in particular areas. There are five genotypes of JEV (I, II, III, IV, and V) currently circulating in the endemic regions. Genotypes IV and V are the oldest whereas I, II, and III are newer. Genotype III JEV was once dominant across all of the South and Southeast Asia and was most frequently isolated in JE-endemic areas until the 1990s. However, a gradual shift from genotype III to I has occurred in many such regions over the last three or four decades ^70, 71^. With this shift in genotype patterns comes a concern of vaccine efficacy. Almost all of the currently available vaccines are developed against genotype III strains of JEV. Even though studies have shown that these are still effective against genotype I ^72^, concerns remain for their effectiveness against genotype V virus ^73^. Thus, to summarize, we can say that the war is still raging and will be continued until JE is defeated.

## Abbreviations

AES, acute encephalitis syndrome; Cas9, clustered regulatory interspaced short palindromic repeat (CRISPR)-associated 9; CNS, central nervous system; DNM2, dynamin 2; EEF1A1, eukaryotic translation elongation factor 1A1; HEK293, human embryonic kidney cell line 293; IL-4, interleukin-4; IVIG, intravenous immunoglobulin; JE, Japanese encephalitis; JEV, Japanese encephalitis virus; MAVS, mitochondrial anti-viral signaling protein; miRNA, microRNA; NFκB, nuclear factor kappa B; NS, non-structural; RIG-I, retinoic acid-inducible gene 1; SHIP1, Src homology 2-containing inositol phosphatase 1; SOCS, suppressor of cytokine signaling; STING, stimulator of interferon genes; TRIM, tripartite motif-containing; WHO, World Health Organization.

## Supplementary Table 1

List of drugs/compounds that have been found to be effective against JEV infection.
